# Human extraintestinal pathogenic *Escherichia coli* strains differ in prevalence of virulence factors, phylogroups, and bacteriocin determinants

**DOI:** 10.1186/s12866-016-0835-z

**Published:** 2016-09-20

**Authors:** Lenka Micenková, Juraj Bosák, Martin Vrba, Alena Ševčíková, David Šmajs

**Affiliations:** 1 Department of Biology, Faculty of Medicine, Masaryk University, Kamenice 5, Building A6, 625 00 Brno, Czech Republic; 2 Department of Clinical Microbiology, Faculty Hospital Brno, Jihlavská 20, 625 00 Brno, Czech Republic

**Keywords:** *Escherichia coli*, ExPEC, Colicin, Microcin, Virulence factor, Bacteriocinogeny

## Abstract

**Background:**

The study used a set of 407 human extraintestinal pathogenic *E. coli* strains (ExPEC) isolated from (1) skin and soft tissue infections, (2) respiratory infections, (3) intra-abdominal infections, and (4) genital smears. The set was tested for bacteriocin production, for prevalence of bacteriocin and virulence determinants, and for phylogenetic typing. Results obtained from the group of ExPEC strains were compared to data from our previously published analyses of 1283 fecal commensal *E. coli* strains.

**Results:**

The frequency of bacteriocinogeny was significantly higher in the set of ExPEC strains (63.1 %), compared to fecal *E. coli* (54.2 %; *p* < 0.01). Microcin producers and microcin determinants dominated in ExPEC strains, while colicin producers and colicin determinants were more frequent in fecal *E. coli* (*p* < 0.01). Higher production of microcin M and lower production of microcin B17, colicin Ib, and Js was detected in the set of ExPEC strains. ExPEC strains had a significantly higher prevalence of phylogenetic group B2 (52.6 %) compared to fecal *E. coli* strains (38.3 %; *p* < 0.01).

**Conclusions:**

Human ExPEC strains were shown to differ from human fecal strains in a number of parameters including bacteriocin production, prevalence of several bacteriocin and virulence determinants, and prevalence of phylogenetic groups. Differences in these parameters were also identified within subgroups of ExPEC strains of diverse origin. While some microcin determinants (mM, mH47) were associated with virulent strains, other bacteriocin types (mB17, Ib, and Js) were associated with fecal flora.

**Electronic supplementary material:**

The online version of this article (doi:10.1186/s12866-016-0835-z) contains supplementary material, which is available to authorized users.

## Background

Extraintestinal pathogenic *E. coli* (ExPEC) strains colonize various sites in the human body and cause diverse extraintestinal infections [1, 2]. Compared to commensal *E. coli* strains, extraintestinal strains have larger genomes and encode more virulence factors [3]. The group of ExPEC strains includes uropathogenic *E. coli* strains, septicemia-associated *E. coli*, meningitis-associated *E. coli,* and other strains [4]. ExPEC strains typically encode i) virulence factors that allow them to bind to human cells (e.g. P-fimbriae, S-fimbriae) [5, 6], ii) factors important for survival in the human body (e.g. siderophores), and iii) factors capable of damaging human cells and tissues (e.g. hemolysin, cytotoxin necrotizing factor) [7].

Another important feature of pathogenic and also commensal *E. coli* strains is production of bacteriocins [8–12]. Colicins and microcins (bacteriocins) are antibacterial proteins or peptides, respectively, differing in a number of characteristics including molecular mass, presence of post-translational modifications, export from producer cells, etc. Colicin Js is known to share features of both colicins and microcins [9, 10, 13, 14]. Bacteriocin production has been shown to play an important ecological role in bacterial competition [15]. In addition to antimicrobial activity, several bacteriocin types have also been shown to inhibit proliferation of eukaryotic cells [16]. In previous reports, an association of bacteriocin production with *E. coli* virulence factors was described [10–12, 17] as well as a positive correlation between the frequency of bacteriocinogeny and the number of virulence factors encoded by *E. coli* strains [17]. In a former study, production of virulence factors typical for the ExPEC pathotype (*sfa*, *pap*, *aer*, *iucC*, *cnf1*, *α-hly*), in the set of fecal *E. coli* strains, was associated with a higher frequency of bacteriocinogeny, a higher prevalence of bacteriocin multi-producers, and a greater abundance of microcins H47, M, V, B17, and colicins E1, Ia, and S4 [17]. In addition, production of microcin types H47, M, I47, E492, and V, and colicin E1 was associated with the uropathogenic *E. coli* pathotype [10–12]. Higher production of microcin V was also detected in septicemia-associated ExPEC strains isolated from blood [18].

In this study, we characterized a set of 407 human extraintestinal pathogenic *E. coli* isolated from different body sites and determined the prevalence of 30 bacteriocin determinants representing most of the known bacteriocin types. In addition, we determined 18 virulence determinants typical of intestinal and extraintestinal pathogenic *E. coli* strains and also the main phylogroups of *E. coli* (A, B1, B2, and D)*.* ExPEC strains were isolated from skin and soft tissue infections, as well as from respiratory, intra-abdominal, and genital infections. Results obtained in this study were compared to a previously characterized and published set of 1283 human fecal *E. coli* strains [19, 20].

## Results

### Origin of extraintestinal pathogenic *E. coli* and fecal strains

The ExPEC strains (*n* = 407) were isolated between 2007 and 2012 from patients attending the University Hospital in Brno, Czech Republic (Table 1 and Additional file 1: Table S1) and characteristics of these strains are shown in Table 1. The ExPEC strains were isolated from patients suffering from skin and/or soft tissue infections (SSTIs) (*n* = 154), from patients with respiratory infections (*n* = 111), from patients with intra-abdominal infections (*n* = 87), and from patients with genital infections (*n* = 55).

**Table 1:** ExPEC strains (*n* = 407) characterized in this study

	Origin of ExPEC strains (*n* = 407)				
Characteristics of patients	SSTIs^a^ (*n* = 154)	Respiratory infections (*n* = 111)	Intra-abdominal infections (*n* = 87)	Genital smears (*n* = 55)	Total no. of strains (*n* = 407)
Females	62	43	46	40	192
Males	92	68	41	15	215
Average age	45.8	41.0	56.4	33.5	45.1
Age range	1y – 98y	1y – 88y	1y – 88y	1y – 76y	1y – 98y

^a^ExPEC strains isolated from patients suffering from skin and/or soft tissue infections

A set of 1283 *E. coli* strains of fecal origin had been isolated from patients in the Czech Republic during the same years (i.e. 2007–2012) and previously described in detail [19, 20].

### Detection of virulence factors in ExPEC and fecal strains

**Fig. 1:**
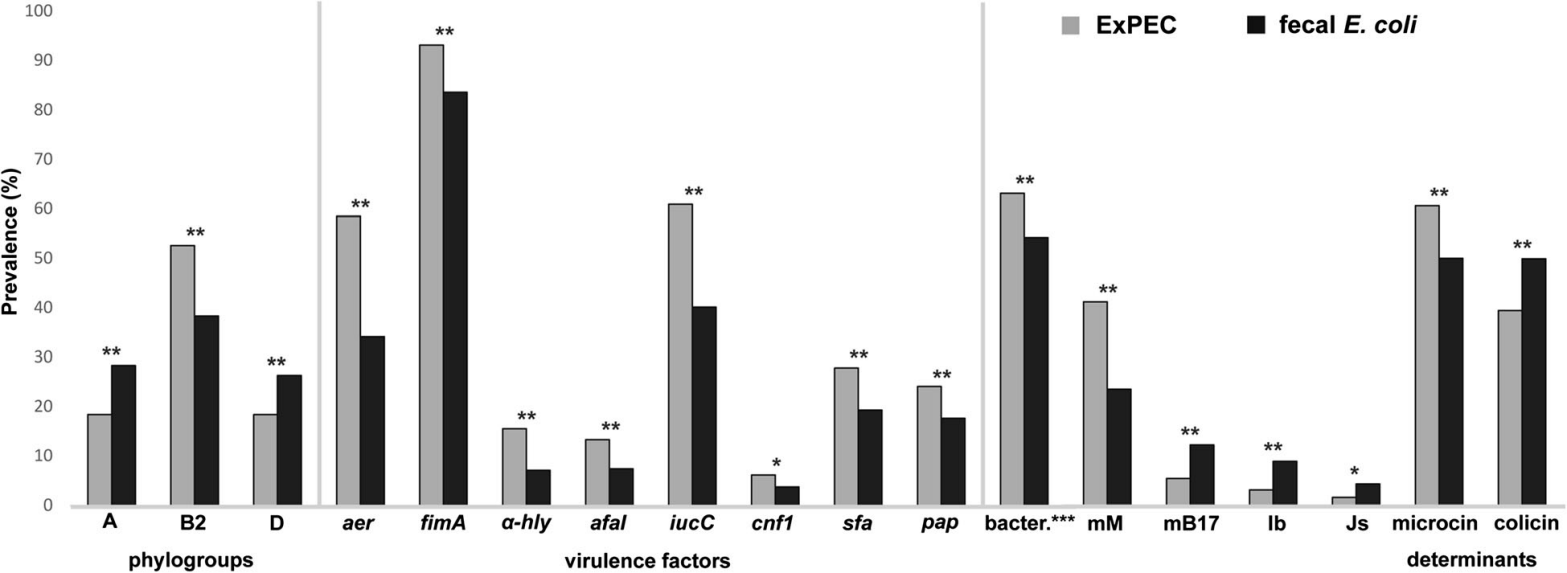
Significant differences in the prevalence of virulence determinants, *E. coli* phylogroups, bacteriocin producers, and the prevalence of bacteriocin determinants among ExPEC and fecal strains (for all results see Additional file 3: Table S3). Note: statistically significant results with *0.05 > *p* > 0.01 or with **p < 0.01; ***bacteriocin producers

Results of detection of 18 DNA determinants (pCVD432*, α-hly*, *afaI*, *aer*, *cnf1*, *sfa*, *pap*, *ial*, *lt*, *st*, *bfpA, eaeA, ipaH, iucC, fimA, stx1*, *stx2*, and *ehly*) encoding 17 different virulence factors in both ExPEC and fecal strains are shown in Fig. 1 and Additional file 2: Table S2. Virulence genes encoding aerobactin synthesis (*iucC*, *aer*), fimbriae type 1 (*fimA*), S (*sfa*) and P (*pap*), afimbrial adhesin I (*afaI*), cytotoxic necrotizing factor (*cnf1*), and α-hemolysin (α-hly) were significantly more common in the set of ExPEC strains, compared to fecal *E. coli* strains (Fig. 1). No virulence determinant was found to be more common among fecal *E. coli* strains, compared to ExPEC strains.

### Distribution of *E. coli* phylogenetic groups in ExPEC and fecal strains

Among 407 tested ExPEC strains, the most prevalent phylogenetic group was B2 (*n* = 214; 52.6 %), followed by phylogroup D (*n* = 75; 18.4 %), A (*n* = 75; 18.4 %), and B1 (*n* = 43; 10.6 %). Compared to fecal *E. coli* strains, ExPEC strains had a higher prevalence of phylogenetic group B2 (38.3 vs. 52.6 %, respectively; *p* < 0.01) (Fig. 1 and Additional file 2: Table S2).

### Bacteriocinogeny and bacteriocin types in ExPEC and fecal strains

The overall frequency of bacteriocinogeny was significantly higher in the set of ExPEC strains (63.1 %), compared to fecal *E. coli* strains (54.2 %; *p* < 0.01) (Fig. 1). While strains producing colicins, but no microcins, were under-represented among ExPEC bacteriocinogenic strains compared to fecal bacteriocinogenic strains (20.2 vs. 28.8 %, respectively; *p* < 0.01), strains producing only microcins (i.e., no colicin types) were more prevalent (45.1 vs. 30.8 %, respectively; *p* < 0.01).

The distribution of bacteriocin determinants encoding different bacteriocin types was different between ExPEC and fecal strains. Among ExPEC bacteriocinogenic strains, a higher prevalence of the microcin M determinant (41.2 vs. 23.5 %, respectively; *p* < 0.01) and a lower prevalence of determinants encoding microcin B17 (5.4 vs. 12.2 %, respectively; *p* < 0.01), colicins Ib (3.1 vs. 8.9 %; respectively; *p* < 0.01), and Js (1.6 vs. 4.3 %, respectively; *p* = 0.05) was detected, compared to fecal *E. coli* (Fig. 1 and Additional file 2: Table S2).

In general, microcin determinants were more prevalent, while colicin determinants were less prevalent among ExPEC strains compared to fecal strains (Fig. 1 and Additional file 2: Table S2).

### Detection of individual bacteriocin determinants in phylogroups of ExPEC and fecal strains

In ExPEC strains, there was a 64.4 % frequency of bacteriocinogeny in the phylogenetic groups A + B1 and 62.6 % in the phylogroups B2 + D. In fecal strains, the frequency of bacteriocinogeny was significantly lower (*p* = 0.03) in the less pathogenic phylogroups A + B1 (49.9 %), compared to phylogroups B2 + D (56.5 %). Since previous reports showed that several bacteriocin determinants were associated with *E. coli* phylogroups [20], we tested for the prevalence of bacteriocin determinants within less and more virulent phylogroups (A + B1 vs. B2 + D, respectively, Table 2 and Additional file 3: Table S3). Bacteriocin type mC7 was found to be more common among ExPEC strains of phylogroup A and B1 compared to the same fecal phylogroups. In contrast to microcin C7, the bacteriocin determinant encoding mM was more common in B2 + D phylogroups of ExPEC compared to fecal strains (Table 2). At the same time, microcin B17 was found to be under-represented among ExPEC strains of phylogroups B2 and D.

**Table 2:** Detection of individual bacteriocin determinants in ExPEC and fecal bacteriocin producers within less virulent phylogenetic groups A + B1 compared to more virulent phylogroups B2 + D

Bacteriocin determinant^a^	ExPEC strains	Fecal strains	*P* value^a^	ExPEC strains	Fecal strains	*P* value
	A + B1 (*n* = 118) %	A + B1 (*n* = 455) %		B2 + D (*n* = 289) %	B2 + D (*n* = 828) %	
Bacteriocinogeny	76 (64.4)	227 (49.9)	0.03	181 (62.6)	468 (56.5)	-
mM determinant^b^	13 (17.1)	24 (10.6)	-	93 (51.4)	139 (29.7)	<0.01
mB17 determinant	4 (5.3)	19 (8.4)	-	10 (5.5)	66 (14.1)	<0.01
mC7 determinant	6 (7.9)	4 (1.8)	0.02	4 (2.2)	8 (1.7)	-

^a^Only statistically significant results are shown, for other results see Additional file 4: Table S4

^b^Bacteriocin determinants (mM, mB17, and mC7) of bacteriocinogenic *E. coli* isolates

### Virulence factors, *E. coli* phylogroups and bacteriocinogeny within ExPEC strains

**Fig. 2:**
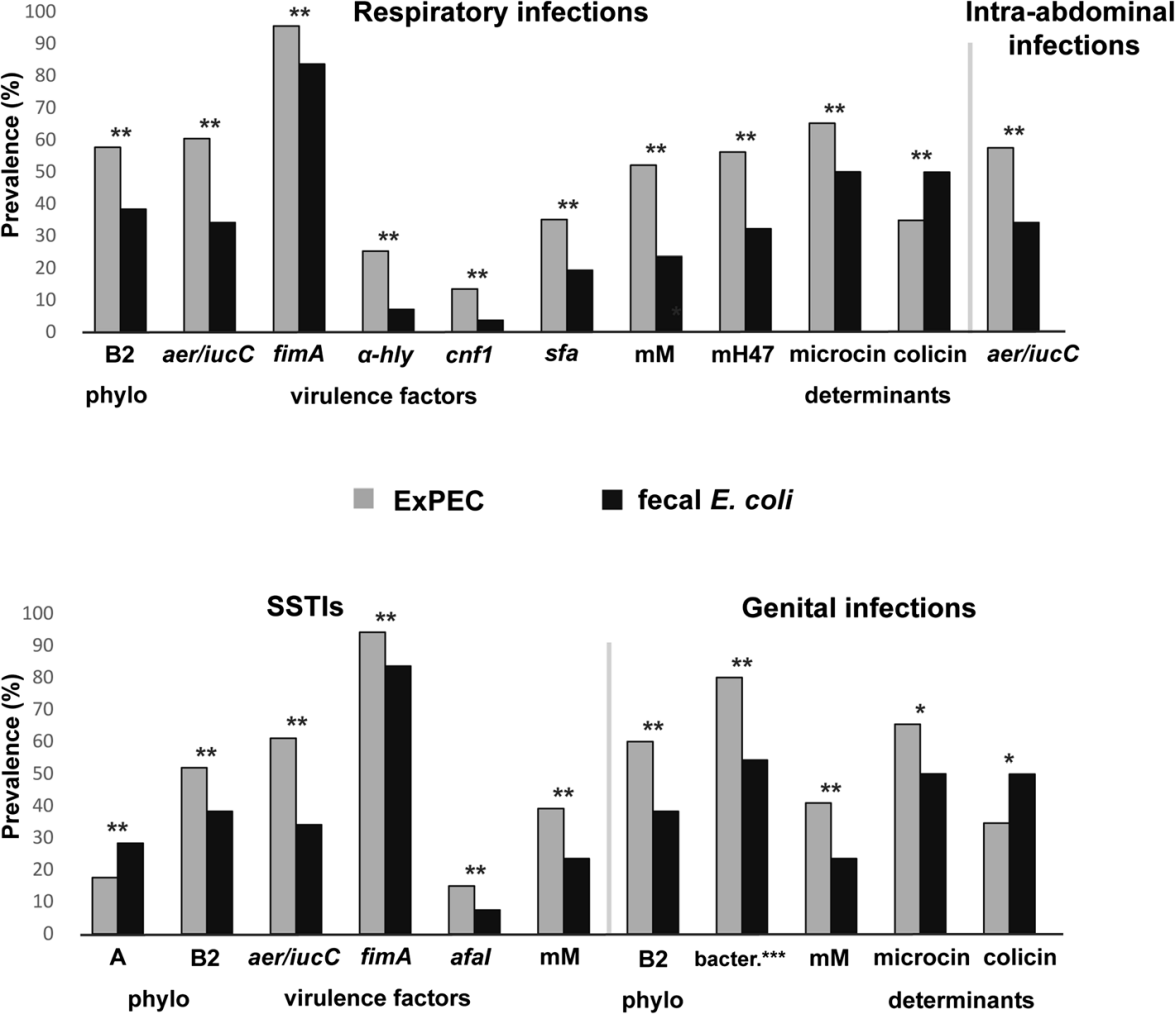
Significant differences in the distribution of virulence factors, *E. coli* phylogroups, bacteriocin production, and bacteriocin determinants between subgroups of ExPEC and fecal strains (for all results see Additional file 4: Table S4). Note: statistically significant results with *0.05 > *p* > 0.01 or with **p < 0.01; ***bacteriocin producers

Compared to fecal *E. coli* strains, ExPEC strains isolated from patients suffering from skin and/or soft tissue infections (SSTIs) (*n* = 154) showed higher prevalence of determinants encoding aerobactin synthesis and uptake, fimbriae type I, and afimbrial adhesin I (Fig. 2 and Additional file 4: Table S4). The ExPEC strains from patients with respiratory infections (*n* = 111) showed a higher prevalence of determinants encoding aerobactin synthesis and uptake, fimbriae type I, α-hemolysin, cytotoxic necrotizing factor, and S-fimbriae. ExPEC from patients with intra-abdominal infections (*n* = 87) had a higher prevalence of determinants encoding aerobactin synthesis and uptake. ExPEC from patients with genital infections (*n* = 55) showed no detectable differences in encoded virulence factors compared to fecal strains (Fig. 2 and Additional file 4: Table S4).

ExPEC strains from SSTIs, strains from respiratory infections, and strains from genital infections, but not strains from intra-abdominal infections, showed a higher prevalence of phylogroup B2 (*p* < 0.01, Fig. 2) compared to fecal strains. In addition, the set of strains isolated from SSTIs also had a lower prevalence of phylogenetic group A (*p* < 0.01) compared to fecal *E. coli* strains (Fig. 2).

Within ExPEC strains, the highest rate of bacteriocinogeny was found in *E. coli* strains isolated from genital smears (80.0 %). Compared to fecal strains, strains isolated from SSTIs and strains from genital infections showed a higher prevalence of the mM determinant. Strains from respiratory infections showed a higher prevalence of determinants encoding both mM and mH47 (Fig. 2). Both, ExPEC strains from respiratory infections, and strains from genital infections, showed a higher prevalence of microcin determinants, respectively (*p* < 0.01). At the same time, these strains showed a lower prevalence of colicin determinants.

## Discussion

ExPEC strains characterized in this study were isolated from various sites of infection including skin and/or soft tissue infections, respiratory, intra-abdominal, and genital infections. Compared to fecal strains isolated during the same time period in a similar geographical area, ExPEC strains showed a higher prevalence of seven known virulence factors. These virulence factors comprised adherence factors, aerobactin synthesis and uptake, and cytotoxin synthesis, which is consistent with increased ability to colonize extraintestinal body sites and represents the initial step in the development of extraintestinal infections. These findings are in agreement with previous studies on ExPEC strains [5–7, 21–25].

Phylogenetic analysis revealed that phylogenetic group B2 was dominant in the set of extraintestinal pathogenic *E. coli* tested in this study. A high prevalence of phylogenetic group B2 is typical of more virulent *E. coli* strains [26] and a higher prevalence of phylogenetic group B2 (35.0 %) has been described in *E. coli* strains isolated from bacteremia [27] and in uropathogenic strains (55.0 %) [28]. In a different set of *E. coli* strains isolated from blood, wound, swab, pus, urine, cerebrospinal fluid, ascitic fluid, and intravascular devices, phylogroups B2 (35.0 %) and D (36.0 %) were found to be most prevalent [29]. Molecular epidemiology, based on MLST (Multilocus sequence typing), also revealed that the phylogroup B2 was correlated with the ExPEC pathotype [23]. ExPEC strains characterized in this study were clearly more virulent compared to fecal strains.

Compared to fecal strains from phylogroup A and B1, microcin type C7 was found to be more common among ExPEC strains of the same phylogroups. These data are consistent with the role of bacteriocin genes regarding increased virulence of *E. coli* strains. Alternatively to bacteriocin genes, genes encoded on the same plasmids could contribute to the increased virulence of these strains since mC7 plasmids are relatively large with a number of genes of predicted and unknown functions [30–33].

A higher frequency of bacteriocin determinant mM among B2 + D phylogroups suggest that microcin M and/or genes in the same linkage group contribute to the virulence of ExPEC strains. Previous reports have shown that the mM and mH47 locus is abundant in uropathogenic isolates, while the cluster seldom appeared in intestinal or in other extraintestinal *E. coli* isolates [10–12, 17, 34]. Our study showed that the percentage of strains coding for mM and mH47 differed among ExPEC strains of diverse origin suggesting that the source of extraintestinal *E. coli* isolates affects the prevalence of mM and mH47 determinants. Despite the fact that microcins H47 and M are typically produced together, sequence analyses have revealed frequent rearrangements at this locus [7, 17, 20, 27, 34]. The incomplete linkage of the microcin H47 and M locus is also supported by our results.

*E. coli* strains isolated from intra-abdominal infections were not phylogenetically distinct from fecal flora suggesting that any *E. coli* strain having access to the abdominal cavity can cause an infection. This was also true for all other tested parameters with just one exception, which related to increased synthesis and uptake of siderophore aerobactin. Access to iron thus appears to be of importance to *E. coli* strains causing infections in intra-abdominal environment

*E. coli* strains isolated from respiratory infections showed a lower number of colicin producers and a higher number of microcin producers, and also a higher representation of phylogroup B2. Unlike other tested ExPEC strains, these strains also showed a higher prevalence of microcin H47, in addition to a higher prevalence of microcin M determinants, suggesting selection for a complete chromosomal region encoding both microcins H47 and M [31].

Strains isolated from genital infections showed the highest rate of bacteriocinogeny (reaching 80 %) and also of microcin production, even though they produced only six different colicin types. Based on the prevalence of phylogroup B2, *E. coli* strains isolated from genital smears were the most virulent in our set of ExPEC strains. Although, there was no evidence for a direct association between ExPEC strains and bacteriocinogeny, previous studies have found an association between virulence factors and bacteriocin determinants, suggesting a role for bacteriocin in bacterial virulence [10–12, 17]. It is widely accepted that strains causing genital infections and uropathogenic strains originate from fecal flora, which represents a reservoir of these strains [35, 36]. Genital infections appear to be caused by only the most virulent and bacteriocinogenic subset of fecal *E. coli* strains.

*E. coli* strains isolated from skin and soft tissue infections, i.e., decubiti, abscesses, and surgical wounds, showed a higher prevalence of microcin M and a higher prevalence of phylogenetic group B2. Results published by Petkovšek et al. (2009) revealed a similarly higher incidence of phylogroup B2 in a group of 102 SSTIs strains [22]. However, in general, *E. coli* strains isolated from skin and soft tissue infections showed a bacteriocinogeny frequency comparable to fecal *E. coli*. Bacteriocin synthesis, therefore, in these skin and soft tissue infections does not appear to be of selective advantage. At the same time, skin and soft tissue infections including decubiti, abscesses, and surgical wounds are typical for hospitalized patients and therefore likely hospital-acquired [22]. It has been previously reported that uropathogenic ESBL-producing hospital-acquired strains showed a low frequency of bacteriocinogeny [37]. Since hospital-acquired strains are often of clonal origin, we have tested genetic heterogeneity among the SSTIs strains used in this study. Detection of four *E. coli* phylogroups, 18 different virulence, and 30 different bacteriocin determinants among the 154 strains analyzed in this study revealed 103 distinct phenotypes (data not shown) suggesting that the strains causing SSTIs are not of clonal origin.

## Conclusions

In summary, we have described a higher frequency of bacteriocinogeny in a set of ExPEC strains isolated from various human extraintestinal infections relative to fecal *E. coli*. Production of bacteriocins is important for most extraintestinal strains isolated from various locations on and in the human body. In general, the extraintestinal environment appears to select for strains with chromosomally encoded microcins (predominantly of mH47 and mM), while plasmid-encoded microcin and colicin types (e.g. mB17, Ia, M) appear to be contra-selected among ExPEC strains suggesting their contribution to fitness in fecal *E. coli* strains.

## Methods

### Bacterial strains

Human extraintestinal *E. coli* strains were collected between 2007 and 2012 from patients attending the University Hospital in Brno (*n* = 407) (Table 1 and Additional file 1: Table S1). From each patient, a single *E. coli* strain was isolated and the ENTEROtest16 (Erba Lachema, Czech Republic) was used for biochemical identification. ExPEC strains used in the study were isolated from various extraintestinal infections and included 1) *E. coli* strains isolated from skin and/or soft tissue infections (SSTIs), 2) *E. coli* strains isolated from respiratory infections (nasal, oral, and throat smears and/or sputum), 3) *E. coli* strains isolated from intra-abdominal infections, and 4) *E. coli* strains isolated from genital smears. ExPEC strains were defined as strains isolated from extraintestinal environment causing an extraintestinal infection (Additional file 1: Table S1).

A set of 1283 *E. coli* strains of fecal origin had been isolated from patients at two university hospitals in Brno (*n* = 1181) and one in Hradec Králové in the Czech Republic during the same years (i.e. 2007–2012) and previously described in detail [19, 20].

For identification of colicin and microcin determinants among tested strains, known bacteriocin producers were used as positive controls: *E. coli* BZB2101pColA - CA31, BZB2102 pColB - K260, BZB2103 pColD - CA23, BZB2107 pColE4 - CT9, BZB2108 pColE5 - 099, BZB2150 pColE6 - CT14, BZB2120 pColE7 - K317, BZB2279 pColIa - CA53, BZB2202 ColIb - P9, BZB2116 pColK - K235, PAP1 pColM - BZBNC22, BZB2123 pColN - 284 (original source: A. P. Pugsley), *E. coli* 189BM pColE2 - P9 (B. A. D. Stocker), *E. coli* 385/80 pColE1, pColV (H. Lhotová), *E. coli* 185 M4 pColE3 - CA38 (P. Fredericq), *E. coli* W3110 pColE8, W3110 pColE9 (J. R. James), *E. coli* K-12 pColS4 (D. Šmajs), *S. boydii* M592 (serovar 8) pColU (V. Horák), *E. coli* K339 pColY (D. Friedman), *Shigella sonnei* (colicinotype 7) pColJs (J. Šmarda), *E. coli* pCol5, *E. coli* pCol10 (H. Pilsl), *E. coli* 449/82 pColX (microcin B17), *E. coli* 313/66 pColG (microcin H47), *E. coli* 363/79 pColV (microcin V, original source: H. Lhotová), *E. coli* TOP10F' pDS601 (microcin C7), *E. coli* D55/1 (microcin J25), and *E. coli* B1239 (microcin L, D. Šmajs) [11, 17].

Positive controls for detection of virulence determinants were taken from our laboratory stock and comprised the following strains: *E. coli* B2917 (pCVD432), *E. coli* B3428 (*α-hly*), *E. coli* B3406 (*afaI*), *E. coli* B3427 (*aer*), *E. coli* B3410 (*cnf1*), *E. coli* B3418 (*sfa*), *E. coli* B3406 (*pap*), *E. coli* B3430 (*ial*), *E. coli* B2541 (*st*), *E. coli* B2802 (*lt*), *E. coli* B1804 (*bfpA*), *E. coli* B2905 (*eaeA*), *E. coli* B2987 (*ipaH*), *E. coli* B3411 (*iucC*), *E. coli* B3404 (*aer*), *E. coli* B3423 (*fimA*), and *E. coli* B2871 (*ehly*).

### Detection of colicin and microcin determinants

A previously described method [11, 17] was used for detection of bacteriocin producers. Briefly, bacteriocinogeny of ExPEC strains (*n* = 407) was tested on agar plates against six indicator strains *E. coli* K12-Row, C6 (ϕ), B1, P400, S40, and *Shigella sonnei* 17. Identification of genetic determinants encoding 23 colicin (A, B, D, E1, E2-9, Ia, Ib, Js, K, L, M, N, S4, U, Y, and 5/10) and 7 microcin (H47, M, B17, C7, J25, L, and V) types, in bacteriocin producers, was performed using the DNA-PCR and colony PCR method. The list of primer pairs and the length of PCR products are in a separate file (Additional file 5: Table S5). The PCR protocol was as follows: 94 °C (2 min for DNA-PCR method; 5 min for colony PCR); 94 °C (30 s), 60 °C (30 s), 72 °C (1 min), 30 cycles; 72 °C (7 min). Because of the sensitivity of microcin types H47 and M to chloroform vapors, all investigated ExPEC and fecal *E. coli* strains were tested for the presence of microcin encoding genes using PCR [38]. PCR products of related bacteriocin types (colicins E2-9, Ia-Ib, and U-Y) were sequenced using dideoxy chain terminator sequencing with amplification primers (Additional file 5: Table S5) and sequences were analyzed using Lasergene software (DNASTAR, Inc., Madison, WI).

### Detection of virulence factors

The presence of 18 virulence determinants (pCVD432*, α-hly*, *afaI*, *aer*, *cnf1*, *sfa*, *pap*, *ial*, *lt*, *st*, *bfpA, eaeA, ipaH, iucC, fimA, stx1*, *stx2*, and *ehly*) encoding 17 virulence factors was screened in ExPEC strains. The primer pair sequences, PCR product lengths and PCR protocols used, were previously described [39–46].

### Phylogenetic group analysis

The phylogenetic groups (A, B1, B2, and D) of 407 extraintestinal *E. coli* strains were determined using the triplex PCR protocol (detection of *chuA*, *yjaA* genes, and TspE4.C2 fragment) described previously [47].

### Statistical analysis

The statistical significance of the prevalence of bacteriocin determinants, *E. coli* phylogenetic groups, and virulence factors was performed by applying standard methods derived from the binomial distribution, including the two-tailed test. *STATISTICA* version 8.0 (StatSoft, Tulsa, OK, USA) was used for statistical calculations. The Bonferroni correction was used in analyses involving multiple comparisons.

## Additional files

Additional file 1:
**Table S1.** Complete list of ExPEC strains (*n* = 407) characterized in this study. (XLSX 90 kb) Additional file 2:
**Table S2.** Distribution of *E. coli* phylogroups, virulence factors, bacteriocin producers, and the prevalence of bacteriocin determinants in ExPEC strains and fecal *E. coli* strains. (XLSX 13 kb) Additional file 3:
**Table S3.** Detection of individual bacteriocin determinants in ExPEC and fecal bacteriocin producers within less virulent phylogenetic groups A + B1 compared to more virulent phylogroups B2 + D. (XLSX 11 kb) Additional file 4:
**Table S4.** Differences in the distribution of virulence factors, *E. coli* phylogroups, bacteriocin production, and bacteriocin determinants between subgroups of ExPEC strains and fecal strains. (XLSX 14 kb) Additional file 5:
**Table S5.** List of primers and the length of PCR products. (DOCX 19 kb)

## Abbreviations

*E. coli*: *Escherichia coli*
ExPEC: Extraintestinal pathogenic *Escherichia coli*
PCR: Polymerase chain reaction
SSTIs: Skin and/or soft tissue infections
MLST: Multilocus sequence typing
ESBL: Extended Spectrum ß-Lactamase
TY agar: Tryptone yeast agar

## References

[1] Orskov I, Orskov F (1985). *Escherichia coli* in extra-intestinal infections. J Hyg (Lond).

[2] Eisenstein BI, Jones GW (1988). The spectrum of infections and pathogenic mechanisms of *Escherichia coli*. Adv Intern Med.

[3] Rasko DA, Rosovitz MJ, Myers GS, Mongodin EF, Fricke WF, Gajer P, Crabtree J, Sebaihia M, Thomson NR, Chaudhuri R, Henderson IR, Sperandio V, Ravel J (2008). The pangenome structure of *Escherichia coli*: comparative genomic analysis of *E. coli* commensal and pathogenic isolates. J Bacteriol.

[4] Johnson JR, Russo TA (2002). Extraintestinal pathogenic *Escherichia coli*: “the other bad *E. coli*”. J Lab Clin Med.

[5] Leffler H, Svanborg-Edén C (1981). Glycolipid receptors for uropathogenic *Escherichia coli* on human erythrocytes and uroepithelial cells. Infect Immun.

[6] Mulvey MA (2002). Adhesion and entry of uropathogenic *Escherichia coli*. Cell Microbiol.

[7] Johnson JR (1991). Virulence factors in *Escherichia coli* urinary tract infection. Clin Microbiol Rev.

[8] Šmarda J, Šmajs D (1998). Colicins - exocellular lethal proteins of *Escherichia coli*. Folia Microbiol (Praha).

[9] Cascales E, Buchanan SK, Duché D, Kleanthous C, Lloubès R, Postle K, Riley M, Slatin S, Cavard D (2007). Colicin biology. Microbiol Mol Biol Rev MMBR.

[10] Azpiroz MF, Poey ME, Laviña M (2009). Microcins and urovirulence in *Escherichia coli*. Microb Pathog.

[11] Šmajs D, Micenková L, Šmarda J, Vrba M, Ševčíková A, Vališová Z, Woznicová V (2010). Bacteriocin synthesis in uropathogenic and commensal *Escherichia coli*: colicin E1 is a potential virulence factor. BMC Microbiol.

[12] Budič M, Rijavec M, Petkovšek Z, Zgur-Bertok D (2011). *Escherichia coli* bacteriocins: antimicrobial efficacy and prevalence among isolates from patients with bacteraemia. PLoS One.

[13] Šmajs D, Weinstock GM (2001). Genetic organization of plasmid ColJs, encoding colicin Js activity, immunity, and release genes. J Bacteriol.

[14] Šmajs D, Weinstock GM (2001). The iron- and temperature-regulated cjrBC genes of *Shigella* and enteroinvasive *Escherichia coli* strains code for colicin Js uptake. J Bacteriol.

[15] Riley MA, Gordon DM (1999). The ecological role of bacteriocins in bacterial competition. Trends Microbiol.

[16] Cornut G, Fortin C, Soulières D (2008). Antineoplastic properties of bacteriocins: revisiting potential active agents. Am J Clin Oncol.

[17] Micenková L, Štaudová B, Bosák J, Mikalová L, Littnerová S, Vrba M, Ševčíková A, Woznicová V, Šmajs D (2014). Bacteriocin-encoding genes and ExPEC virulence determinants are associated in human fecal *Escherichia coli* strains. BMC Microbiol.

[18] Davies DL, Falkiner FR, Hardy KG (1981). Colicin V production by clinical isolates of *Escherichia coli*. Infect Immun.

[19] Kohoutová D, Šmajs D, Moravková P, Cyrany J, Moravková M, Forstlová M, Čihák M, Rejchrt S, Bureš J (2014). *Escherichia coli* strains of phylogenetic group B2 and D and bacteriocin production are associated with advanced colorectal neoplasia. BMC Infect Dis.

[20] Micenková L, Bosák J, Štaudová B, Kohoutová D, Čejková D, Woznicová V, Vrba M, Ševčíková A, Bureš J, Šmajs D (2016). Microcin determinants are associated with B2 phylogroup of human fecal *Escherichia coli* isolates. Microbiol Open.

[21] Mokady D, Gophna U, Ron EZ (2005). Virulence factors of septicemic *Escherichia coli* strains. Int J Med Microbiol.

[22] Petkovsek Z, Elersic K, Gubina M, Zgur-Bertok D, Starcic Erjavec M (2009). Virulence potential of *Escherichia coli* isolates from skin and soft tissue infections. J Clin Microbiol.

[23] Köhler CD, Dobrindt U (2011). What defines extraintestinal pathogenic *Escherichia coli*?. Int J Med Microbiol.

[24] Fakruddin M, Mazumdar RM, Chowdhury A, Mannan KS (2013). A preliminary study on virulence factors & antimicrobial resistance in extra-intestinal pathogenic *Escherichia coli* (ExPEC) in Bangladesh. Indian J Med Res.

[25] Koga VL, Tomazetto G, Cyoia PS, Neves MS, Vidotto MC, Nakazato G, Kobayashi RK (2014). Molecular screening of virulence genes in extraintestinal pathogenic *Escherichia coli* isolated from human blood culture in Brazil. Biomed Res Int.

[26] Picard B, Garcia JS, Gouriou S, Duriez P, Brahimi N, Bingen E, Elion J, Denamur E (1999). The link between phylogeny and virulence in *Escherichia coli* extraintestinal infection. Infect Immun.

[27] Santos AC, Zidko AC, Pignatari AC, Silva RM (2013). Assessing the diversity of the virulence potential of *Escherichia coli* isolated from bacteremia in São Paulo, Brazil. Braz J Med Biol Res.

[28] Mirzarazi M, Rezatofighi SE, Pourmahdi M, Mohajeri MR (2015). Occurrence of genes encoding enterotoxins in uropathogenic *Escherichia coli* isolates. Braz J Microbiol.

[29] Chakraborty A, Saralaya V, Adhikari P, Shenoy S, Baliga S, Hegde A (2014). Characterization of *Escherichia coli* Phylogenetic Groups Associated with Extraintestinal Infections in South Indian Population. Indian J Pathol Microbiol.

[30] Waters VL, Crosa JH (1991). Colicin V virulence plasmids. Microbiol Rev.

[31] Duquesne S, Destoumieux-Garzón D, Peduzzi J, Rebuffat S (2007). Microcins, gene-encoded antibacterial peptides from enterobacteria. Nat Prod Rep.

[32] Jeziorowski A, Gordon DM (2007). Evolution of microcin V and colicin Ia plasmids in *Escherichia coli*. J Bacteriol.

[33] Šmajs D, Strouhal M, Matějková P, Čejková D, Cursino L, Chartone-Souza E, Šmarda J, Nascimento AM (2008). Complete sequence of low-copy-number plasmid MccC7-H22 of probiotic *Escherichia coli* H22 and the prevalence of mcc genes among human *E. coli*. Plasmid.

[34] Gordon DM, O’Brien CL (2006). Bacteriocin diversity and the frequency of multiple bacteriocin production in *Escherichia coli*. Microbiology.

[35] Nielsen KL, Dynesen P, Larsen P, Frimodt-Møller N (2014). Faecal *Escherichia coli* from patients with *E. coli* urinary tract infection and healthy controls who have never had a urinary tract infection. J Med Microbiol.

[36] Moreno E, Andreu A, Pigrau C, Kuskowski MA, Johnson JR, Prats G (2008). Relationship between *Escherichia coli* strains causing acute cystitis in women and the fecal *E. coli* population of the host. J Clin Microbiol.

[37] Micenková L, Šišková P, Bosák J, Jamborová I, Černohorská L, Šmajs D (2014). Characterization of human uropathogenic ESBL-producing *Escherichia coli* in the Czech Republic: spread of CTX-M-27-producing strains in a university hospital. Microb Drug Resist.

[38] Patzer SI, Baquero MR, Bravo D, Moreno F, Hantke K (2003). The colicin G, H and X determinants encode microcins M and H47, which might utilize the catecholate siderophore receptors FepA, Cir, Fiu and IroN. Microbiology (Reading, Engl).

[39] Martínez JL, Herrero M, de Lorenzo V (1994). The organization of intercistronic regions of the aerobactin operon of pColV-K30 may account for the differential expression of the iucABCD iutA genes. J Mol Biol.

[40] Schmidt H, Knop C, Franke S, Aleksic S, Heesemann J, Karch H (1995). Development of PCR for screening of enteroaggregative *Escherichia coli*. J Clin Microbiol.

[41] Yamamoto S, Terai A, Yuri K, Kurazono H, Takeda Y, Yoshida O (1995). Detection of urovirulence factors in *Escherichia coli* by multiplex polymerase chain reaction. FEMS Immunol Med Microbiol.

[42] Kuhnert P, Hacker J, Mühldorfer I, Burnens AP, Nicolet J, Frey J (1997). Detection system for *Escherichia coli*-specific virulence genes: absence of virulence determinants in B and C strains. Appl Environ Microbiol.

[43] Paton AW, Paton JC (1998). Detection and characterization of Shiga toxigenic *Escherichia coli* by using multiplex PCR assays for stx1, stx2, eaeA, enterohemorrhagic *E. coli* hlyA, rfbO111, and rfbO157. J Clin Microbiol.

[44] Paciorek J (2002). Virulence properties of *Escherichia coli* faecal strains isolated in Poland from healthy children and strains belonging to serogroups O18, O26, O44, O86, O126 and O127 isolated from children with diarrhoea. J Med Microbiol.

[45] López-Saucedo C, Cerna JF, Villegas-Sepulveda N, Thompson R, Velazquez FR, Torres J, Tarr PI, Estrada-García T (2003). Single multiplex polymerase chain reaction to detect diverse loci associated with diarrheagenic *Escherichia coli*. Emerging Infect Dis.

[46] Bírošová E, Siegfried L, Kmeťová M, Makara A, Ostró A, Gresová A, Urdzík P, Liptáková A, Molokácová M, Bártl R, Valanský L (2004). Detection of virulence factors in alpha-haemolytic *Escherichia coli* strains isolated from various clinical materials. Clin Microbiol Infect.

[47] Clermont O, Bonacorsi S, Bingen E (2000). Rapid and simple determination of the *Escherichia coli* phylogenetic group. Appl Environ Microbiol.

