# Supplementary material for: Human extraintestinal pathogenic Escherichia coli strains differ in prevalence of virulence factors, phylogroups, and bacteriocin determinants
Source: BMC Microbiol. 2016 Sep 20;16:218. doi: 10.1186/s12866-016-0835-z (PMC5028950; doi:10.1186/s12866-016-0835-z)
Supplement: Additional file 5: — Table S5. List of primers and the length of PCR products. (DOCX 19 kb) [file 12866_2016_835_MOESM5_ESM.docx]

**Table S5. List of primers and the length of PCR products**

| **Bacteriocin types and virulence factors** | **Primer name** | **5´-sequence-3´** | **Length** | **Reference of primers** |
| --- | --- | --- | --- | --- |
|  |  |  | **of PCR product (nt)** |  |
| A | ColA-F | cgtggggaaaagtcatcatc |  |  |
|  | ColA-R | gctttgctctttcctgatgc | 475 | [11] |
| B | colicinB-F | aagaaaatgacgagaagacg |  |  |
|  | colicinB-R | gaaagaccaaaggctataagg | 492 | [30] |
| D | ColD-F | ctggactgctgctggtgata |  |  |
|  | ColD-R | gaaggtgcgcctactactgc | 420 | [11] |
| E1 | colicinE1-F | tgtggcatcgggcgagaata |  |  |
|  | colicinE1-R | ctgcttcctgaaaagcctttt | 649 | [30] |
| E1* | cea2F | ggtggaactggaggtagcaa |  |  |
|  | cea2R | acgtcgttgttgttctgcttcct | 357 | [17] |
| E2 | ColE2-F | tgatgctgctgcaaaagag |  |  |
|  | ColE2-R | ttcaaagcgttccctaccac | 409 | [11] |
| E3 | ColE3-F | taagcaggctgcatttgatg |  |  |
|  | ColE3-R | tcggatctggacctttcaac | 413 | [11] |
| E4 | ColE4-F | gaaggctgcatttgatgct |  |  |
|  | ColE4-R | cggatccggacctttaattt | 409 | [11] |
| E5 | ColE3-F | taagcaggctgcatttgatg |  |  |
|  | ColE5-R | ttgaattctcgaatcgtcca | 430 | [11] |
| E6 | ColE6-F | accgaacgtccaggtgtt |  |  |
|  | ColE6-R | tttagcctgtcgctcctgat | 399 | [11] |
| E7 | ColE7-F | gcattctgccatctgaaat |  |  |
|  | ColE7-R | cttctgcccactttctttcg | 431 | [11] |
| E8 | ColE3-F | taagcaggctgcatttgatg |  |  |
|  | ColE8-R | gactgattggcttgtcgtga | 449 | [11] |
| E9 | ColE3-F | taagcaggctgcatttgatg |  |  |
|  | ColE9-R | gacttttctccctccgacct | 418 | [11] |
| Ia | ColIa-F | gcatgcaaatgacgctctta |  |  |
|  | ColIa-R | gaggacgccagttctctgtc | 473 | [11] |
| Ib | ColIb-F | aacgagtgggtcgatgattc |  |  |
|  | ColIb-R | ccttttctgcgctcgtattc | 464 | [11] |
| Js | ColJs-F | tcaaaatgtttgggctcctc |  |  |
|  | ColJs-R | taatctgccctgtcccactg | 254 | [11] |
| K | ColK-F | cagaggtcgctgaacatgaa |  |  |
|  | ColK-R | tccgctaaatcctgagcaat | 469 | [11] |
| L | Col28b(L)-F | tgcatattgaaagcgtcagc |  |  |
|  | Col28b(L)-R | caggttatcccctctcacca | 449 | [17] |
| M | ColM-F | gcttaccacttcgcaaaacc |  |  |
|  | ColM-R | gagcgactctccgataatgc | 429 | [11] |
| N | ColN-F | agcttggcgagtatcttgga |  |  |
|  | ColN-R | caacacagccccgaataaac | 401 | [11] |
| S4 | ColS4-F | tatatggcccaactgctggt |  |  |
|  | ColS4-R | cgtaaggacggacacctgtt | 456 | [11] |
| U | ColU-F | tgattgctgcgagaaaaatg |  |  |
|  | ColU-R | tctgacagcctctccctgtt | 485 | [11] |
| Y | ColY-F | gcaggcagaaaagaacaagg |  |  |
|  | ColY-R | cggacgttatttgccttcat | 477 | [11] |
| 5 | Col5-F | cattggcaaaagcgaaatct |  |  |
|  | Col5-R | tgcaactctggaaacaatcg | 443 | [11] |
| 10 | Col10-F | ggttaccggatttcctggat |  |  |
|  | Col10-R | ttctagatgcttggcccact | 448 | [11] |
| mB17 | microcin B17-F | tcacgccagtctccattaggtgttggcatt |  |  |
|  | microcin B17-R | ttccgccgctgccaccgtttccaccactac | 135 | [30] |
| mC7 | microcin C7-F | cgttcaactgttgcaatgct |  |  |
|  | microcin C7-R | agttgaggggcgtgtaattg | 134 | [11] |
| mH47 | microcin H47-F | cactttcatcccttcggattg |  |  |
|  | microcin H47-R | agctgaagtcgctggcgcacctcc | 227 | [30] |
| mJ25 | microcin J25-F | tcagccatagaaagatataggtgtaccaat |  |  |
|  | microcin J25-R | tgattaagcattttcattttaataaagtgt | 175 | [30] |
| mL | microcin L-F | ggtaaatgatatatgagagaaataacgtta |  |  |
|  | microcin L-R | tttcgctgagttggaatttcctgctgcatc | 233 | [30] |
| mM | M-DS2009-F | cgtttattagcccgggattt |  |  |
|  | M-DS2009-R | gcagacgaagaggcacttg | 166 | [17] |
| mV | microcin V-F | cacacacaaaacgggagctgtt |  |  |
|  | microcin V-R | cttcccgcagcatagttccat | 680 | [30] |
